# Book Reviews

**Published:** 1916-02

**Authors:** 


### Book Reviews.

A Reference Handbook of the Medical Sciences.—Embracing the entire range of Scientific and Practical Medicine and Allied Sciences. By various writers. Third edition, completely revised and rewritten. Edited by Thomas Lathrop Stedman, A. M., M. D. Complete in eight imperial quarto volumes. Volume V, 929 double column pages, illustrated by 733 engravings and six full-page plates in black and colors. Wm. Wood & Co., New York.

The fifth volume of this great undertaking appears with commendable promptness. It contains 929 double column pages; four hundred and eighty-four separate articles, by one hundred and twenty-three writers, all authorities upon their several topics. It is illustrated by seven hundred and thirty-three cuts in the text, and six full-page plates in black and colors. The subjects run from "Head, Wounds of" to "Life Insurance Medicine."

The Reference Handbook is probably so well known that no particular description is required nowadays, the first edition having appeared in 1884. The nick-name often given it, "the doctor's bible," is an indication of its usefulness to the practitioner of medicine. This third edition is fully up to the standard of its predecessors, and it seems hardly conceivable that anyone who could experience its usefulness would not promptly secure it for his convenience of reference.

---

YOUR BABY. By Dr. E. B. Lowry. Forbes & Co., Chicago. Price, $1.00 net.

This is a book which every young mother and prospective mother in the land should read; for it makes motherhood easy and will remove the fears and troubles of many women. Everything which a mother wants to know and must know regarding the care of herself and her baby is clearly told in this helpful book. It shows how to avoid mistakes, what to do and what not to do.

The future of the nation depends upon the babies of today and therefore any book which helps to give a human life a fair start in health should be welcomed and widely circulated. Such a book is this splendid volume by Dr. Lowry. That there is great need for the fullest possible circulation of the book is evident when we consider the appalling fact that nearly half the babies born die before they are a year old, which means that about 300,000 such babies die annually in the United States.

Dr. Lowry not only pleads for better babies but plainly tells how to prepare for them; everything that is essential to the happiness and health of the mother and child is told. Nearly half the book is devoted to the mother's care of herself before the baby comes and this part alone is invaluable to any expectant mother. A very timely chapter considers the various methods offered for painless childbirth and much light is thrown on some fallacies and uncertain methods.

This book contains the latest and best approved methods for the care of the baby,—its feeding, clothing, exercise, sleep and training. It is full of common sense help and facts that many mothers might overlook. Like all Dr. Lowry's books it is permeated with an earnest spirit of helpfulness and wise, sane direction. If a prospective mother can have only one book on the subject of maternity and infancy it should be this safe and practical guide by Dr. Lowry, who is an authority of long experience in the health care of women and children.

---

A Synopsis of Medical Treatment. By George C. Shattuck, M. D. Second revised printing of the second edition. Price, $1.25. W. M. Leonard, Boston, Publisher.

This book presents clearly and concisely sound principles of treatment, based on known pathology. The methods described are selected from those that have been tried at the Massachusetts General Hospital and in private practice. Hence the work sets forth in detail the Postgraduate Course in Clinical Medicine given, at the hospital, by well known teachers.

The first edition was twice printed. In reprinting the second edition opportunity has been used to revise and complete minor details.

The repeated editions and printings of this book indicate its practical value to physicians.

As was stated of the last printing in a review in a prominent journal, "Of particular value is the opportunity which this little handbook affords, of gaining an insight into the important rules of treatment as carried out in one of the largest and oldest hospitals of this country. The worth of the book is out of all proportion to its size."

